# Effects of methylphenidate on attentional set-shifting in a genetic model of attention-deficit/hyperactivity disorder

**DOI:** 10.1186/1744-9081-8-10

**Published:** 2012-02-28

**Authors:** Ai-hua Cao, Lin Yu, Yu-wei Wang, Jun-mei Wang, Le-jin Yang, Ge-fei Lei

**Affiliations:** 1 Department of Pediatrics, Qilu Hospital, Shandong University, Jinan 250012, People's Republic of China; 2 Department of psychology, Qilu Hospital, Shandong University, Jinan 250012, People's Republic of China; 3 Department of Pediatrics, Qilu Hospital, Shandong University, #107 West Wenhua Road, Jinan 250012, People's Republic of China

**Keywords:** Attentional set-shifting, Attention deficit/hyperactivity disorder, Spontaneous hypertensive rat, Methylphenidate

## Abstract

**Background:**

Although deficits of attentional set-shifting have been reported in individuals with attention deficit/hyperactivity disorder (ADHD), it is rarely examined in animal models.

**Methods:**

This study compared spontaneously hypertensive rats (SHRs; a genetic animal model of ADHD) and Wistar-Kyoto (WKY) and Sprague-Dawley (SD) rats (normoactive control strains), on attentional set-shifting task (ASST) performance. Furthermore, the dose-effects of methylphenidate (MPH) on attentional set-shifting of SHR were investigated. In experiment 1, ASST procedures were conducted in SHR, WKY and SD rats of 8 each at the age of 5 weeks. Mean latencies at the initial phase, error types and numbers, and trials to criteria at each stage were recorded. In experiment 2, 24 SHR rats were randomly assigned to 3 groups of 8 each-- MPH-L (lower dose), MPH-H (higher dose), and SHR-vehicle groups. From 3 weeks, they were administered 2.5 mg/kg or 5 mg/kg MPH or saline respectively for 14 consecutive days. All rats were tested in the ASST at the age of 5 weeks.

**Results:**

The SHRs generally exhibited poorer performance on ASST than the control WKY and SD rats. Significant strain effects on mean latency [F (2, 21) = 639.636, *p *< 0.001] and trials to criterion [F (2, 21) = 114.118, *p *< 0.001] were observed. The SHRs were found to have more perseverative and regressive errors than the control strains (*p *< 0.001). After MPH treatment, the two MPH treated groups exhibited significantly longer latency and fewer trials to reach criterion than the SHR-vehicle group and the MPH-L group exhibited fewer trials to reach criterion in more stages compared with the MPH-H group. Significant main effects of treatment [F (2, 21) = 52.174, *p *< 0.001] and error subtype [F (2, 42) = 221.635, *p *< 0.01] were found.

**Conclusions:**

The SHR may be impaired in discrimination learning, reversal learning and attentional set-shifting. Our study provides evidence that MPH may improve the SHR's performance on attentional set-shifting and lower dose is more effective than higher dose.

## Background

Attention deficit/hyperactivity disorder (ADHD), which is characterized by developmentally inappropriate symptoms of inattention, impulsivity and hyperactivity, is among the most common neuropsychiatric disorders in children. The symptoms of ADHD manifest as distractibility, difficulty in sustaining attention, and a failure to appropriately control motor responses. Some have suggested that all of these symptoms can be attributed to a primary deficit of cognition in ADHD sufferers, particularly impairment of executive functions [1]. Previous studies have reported deficits of attentional set-shifting-a type of executive functions-in individuals with ADHD [2-4]. Assessing set-shifting in an animal model can help elucidate the neural mechanisms of ADHD. The spontaneously hypertensive rat (SHR) has been evaluated extensively as an animal model of ADHD, including its neurochemical, genetic, and behavioral characteristics [5]. However, to our knowledge, there are only two studies addressed the nature of set-shifting ability in SHR, which generated conflicting results. Kantak et al. [6] in 2008 found that SHRs exhibited poorer set-shifting compared to inbred Wistar-Kyoto (WKY). Chess AC et al. [7] reported that the SHRs were faster than Wistar rats in shifting to the opposite discrimination when there was 1 or 2 days between the initial discrimination and the shift but equivalent in shifting when the shift between discriminations occurred immediately after a criterion had been met in the first discrimination, which may be due to a failure of SHRs to store or retrieve an initial discrimination and/or latent inhibition over a delay. The divergence of these 2 studies may due to different strains or experimental conditions. Given the importance of set-shifting for understanding ADHD, more studies are needed.

Attentional set-shifting is a form of executive function that represents behavioral flexibility. It requires attending to relevant stimuli while ignoring irrelevant stimuli and subsequently shifting the allocation of attention, either within "dimensions" or between "dimensions" [8]. Deficits of set-shifting in individuals with ADHD have been investigated with some different neuropsychological tests, and the results may depend on which test is administered. For example, in the attentional set-shifting paradigm from the Attentional Battery of the Cambridge Neuropsychological Test Automated Battery (CANTAB), participants are required to discriminate between two stimuli that differ in color and shape. Children and adults with ADHD sometimes make more errors in a set-shifting [9,10] and sometimes do not [11]. On the other hand, the Trail Making Test (TMT), Part B from the Halstead-Reitan battery generally takes longer to complete (an indication of slower set-shifting) [12,13]. The limitations of these studies may also include variability in diagnosis, sample size and demographics. Recently, the attentional set-shifting task (ASST), a similar procedure to commonly used attentional set-shifting testing in humans with ADHD named the Wisconsin Card Sorting Task (WCST) [14], was developed for rats [8] and mice [15] model studies. In ASST, animals are trained in serial discrimination learning stages to acquire a rule and form an attentional set of a stimulus dimension. After that they will be required to shift attention from this dimension to another previously irrelevant stimulus dimension. This ability of shifting is tested as cognitive flexibility (extradimensional shift, ED shift). Meanwhile, other prefrontal cortical dependent cognitive functions including working memory (reversal learning), procedural memory (discrimination learning) can also be measured separately. Assessing attentional set-shifting in an animal model of ADHD can help address some of the limitations of human study and elucidate its neural mechanisms.

Methylphenidate (MPH) is one of the most commonly used psycho-stimulants for the treatment of ADHD, which has been found to improve spatial working memory, response inhibition, and other cognitive functions in children with ADHD [16,17] and corresponding behavior in animal models of ADHD [18]. However, neuropsychological tests of therapeutic effects of MPH on attentional set-shifting in ADHD have yielded inconsistent results (Table 1). The discrepancy of results may come from different treatment regime, different age or sex proportions of participants or lack of appropriate controls. Some studies indicate that MPH produces an inverted U dose response curve on cognitive performance [19]. At lower dose (lower than 5.0 mg/kg for rats), MPH could reduce movement, impulsivity and increase cognitive function. For example, some research show that lower dose of MPH could improve the performance in delayed alternation task, sustained attention task [20,21] and spatial learning and memory task [22] in rats of ADHD models. In contrast, higher dose of MPH (no less than 5.0 mg/kg for rats) may impair cognition, elicit increase in locomotor activity [23], as well as produce side effects like agitation, hallucinations, psychosis, lethargy, seizures, tachycardia, dysrhythmia, hypertension, and hyperthermia in humans of corresponding dosage [24]. Given the discrepancy of results, more studies are needed to identify the effects of MPH on attentional set-shifting in ADHD.

**Table 1:** Studies examining effects of methylphenidate on attentional set-shifting tests in children with ADHD or animal models

Citation	Dose	Time	Outcome	Subjects
[16]	0.5 mg/kg	acute	positive effects of ED	Children with ADHD
[25]	0.25 mg/kg 0.5 mg/kg	6 days	performance were enhanced at a low dose while worsened at a moderate dose of ED	Children with ADHD
[26]	0.3 mg/kg 0.6 mg/kg	acute	no change	Children with ADHD
[27]	35 mg/day	no information	positive effects of ED	Children with ADHD
[28]	0.3 mg/kg 0.6 mg/kg	4 weeks	no change	Children with ADHD
[6]	1.5 mg/kg	28 days	positive effects of ID and ED	SHR

This study aimed to assess the ability of attentional set-shifting in SHRs (genetic animal model of ADHD) compared with WKY and Sprague-Dawley (SD) rats (normoactive control strains) by ASST, which applied us a tool for further investigation of the neurobiological and physiological mechanism underlying ADHD. Furthermore, in Experiment 2 dose effects of MPH on acquiring, maintaining and shifting attentional set of the SHRs were investigated in order to delineate the effects of MPH on attentional set-shifting deficits in ADHD and enhance the understanding of the effects of drug treatment on ADHD.

## Methods

### Subjects

In experiment 1, male rats of the SHR (n = 8), WKY (n = 8) and SD (n = 8) strains were obtained at 3 weeks of age from the Laboratory Animal Co., Lake Hayes Division (Shanghai, China). Rats were housed individually in plastic cages, in a 12 hr light/dark cycle (lights on from 06:00 to 18:00). Food and water were provided ad libitum and the temperature was maintained at 21-23°C. Prior to training on the set-shifting task, weights were obtained and rats were then food deprived to 85% of the free-feeding standard weight. In experiment 2, 24 SHRs were randomly assigned to 3 groups of 8 each: MPH-L (lower dose of MPH), MPH-H (higher dose of MPH) and SHR-vehicle (saline) groups. Rats in the MPH-L group were treated daily with MPH by dose of 2.5 mg/kg [6], in the MPH-H treated with 5 mg/kg [6] MPH, while animals in the SHR-vehicle were treated daily with vehicle (saline). MPH (Ritalin^®^) was obtained from Qilu Hospital of Shandong University and was dissolved in saline. Peritoneal injection was conducted at 8 am daily at the age of 3 weeks for 14 consecutive days. Rats were tested in the ASST at the age of 5 weeks. All animal procedures were conducted in strict compliance with Protocols for Animal Use for Research and Education approved by Medicine Animal Review Committee of Shandong University.

### Apparatus

The attentional set-shifting apparatus and procedures were modifications of those previously described by Birrell and Brown [8]. Rats were trained to retrieve food reward by digging in small cups [29]. The test apparatus was an adapted plastic cage (45 × 30 × 15 cm) with a transparent plexiglass lid. Two equal-sized choice compartments (L and R, 15 × 15 cm) at one end of the apparatus could be accessed through sliding doors from a larger starting compartment (30 × 30 cm) at the other end. A cylindrical food cup (40 mm diameter, 35 mm high) in each choice compartment could be baited with a small piece of cereal (30 mg) as a food reward which was covered with a layer of scented digging medium. The presence or absence of food reward in a cup was indicated by either olfactory stimuli (scent of the digging medium) or tactile stimuli (type of digging medium). We made the digging mediums with according scent by putting the digging mediums and perfume together for 12 hours.

### Habituation

On the day before testing, each rat was given access to the test apparatus for adapting for 1 h. Rats were trained to dig reliably for food reward in the cups. Two unscented cups were placed in the choice compartments and baited, with the reward covered with increasing amounts of home cage bedding. After rats learned to reliably retrieve the rewards from fully baited cups, they were transferred to a series of simple discriminations (SDs) (rubber vs. masking tape; vinegar vs. soy sauce; styrofoam vs. shredded paper) to a criterion of six consecutive correct responses. All rats were trained using the same stimulus exemplars and in the same order. These training stimuli were not used again in later testing sessions.

### Testing paradigm

Following the training day, rats were tested on a series of increasingly difficult discriminations (Table 2). Briefly, a trial was initiated by raising the sliding door to give the rat access to the two cups, only one of which was baited. Response latency from opening the sliding door to the first dig was recorded. Once the rat retrieved the bait, it was allowed to consume the reward before being returned to the starting compartment. In the first 4 trials (exploratory trials), the rat was allowed to dig in both cups, and an error was recorded if the first dug occurred in the unbaited cup. From trial 5 onwards, the rat was allowed to dig in one cup only. If the rat started to dig in the unbaited cup, an error was recorded and the trial was terminated immediately. Testing at each stage continued until the rat reached the criterion of six consecutive correct trials. Trials to criterion and errors to criterion were recorded for each rat and each stage. The test box was wiped down with 70% alcohol at the end of each rat's session to remove odor cues.

**Table 2:** Examples of stimulus pairs used in ASST and the progression through the stages of the ASST

Stages	SD	CD	CDR	ID	IDR	ED	EDR
Series1 (cup1-cup2)	O1▲- O2	O1/M1▲- O2/M2 O1/M2▲-O2/M1	O1/M1- O2/M2▲ O1/M2- O2/M1▲	O3/M3▲- O4/M4 O3/M4▲- O4/M3	O3/M3- O4/M4▲ O3/M4- O4/M3▲	O5/M5▲- O6/M6 O5/M6- O6/M5▲	O5/M5- O6/M6▲ O5/M6▲- O6/M5
Relevant dimension	Odor	Odor	Odor	Odor	Odor	Medium	Medium
Series 2 (cup1-cup2)	M1▲- M2	M1/O1▲- M2/O2 M1/O2▲- M2/O1	M1/O1- M2/O2▲ M1/O2 - M2/O1▲	M3/O3▲- M4/O4 M3/O4▲- M4/O3	M3/O3- M4/O4▲ M3/O4- M4/O3▲	M5/O5▲- M6/O6 M5/O6- M6/O5▲	M5/O5- M6/O6▲ M5/O6▲- M6/O5
Relevant dimension	Medium	Medium	Medium	Medium	Medium	Odor	Odor

The baited cup is signified by ▲. The exemplars were always presented in pairs and varied. The following pairs of exemplars were used: odor: Pair 1: O1- lemon, O2- cinnamon, Pair 2: O3- cloves, O4- rose, Pair 3: O5- peppermint, O6- vanilla; Medium: Pair1: M1-sawdust, M2-woodchips, Pair 2: M3-sand, M4-gravel, Pair 3: M5-confetti, M6- plastic pellets

Table 2 shows the order of stages, which were the same for all rats. In the simple discrimination (SD), the cups differed along only one of the two dimensions, either the odor or the digging medium. For the compound discrimination (CD), an irrelevant second dimension was introduced. The third stage was a reversal of CD (CDR), in which all exemplars and the relevant dimension remained unchanged, but the previously correct stimulus was now incorrect. For the fourth stage called intradimensional shift (ID), new exemplars of both dimensions (new odor and new medium) were used, but the relevant dimension was the same as before. The fifth stage was a reversal of ID (IDR), as in CDR. The sixth stage was extradimensional shift (ED), the most important stage of ASST, in which new exemplars of both dimensions were used and the animal had to shift attention to the previously irrelevant dimension (from odor/medium to medium/odor). The last stage was reversal of ED (EDR).

Errors during testing were broken down into 3 subtypes as has been previously described: perseverative errors, regressive errors and never-reinforced errors [30-34]. Briefly, perseverative errors were scored when a rat continued to use the previously relevant but currently irrelevant rule. In 6 out of every 12 consecutive trials the rat was allowed to respond in this way. To score these types of errors, trials were separated into consecutive blocks of four trials each in which perseverative errors were counted when rats chose unbaited cup 3 or more trials per block. Once a rat made 2 or fewer incorrect choice during a block, all subsequent perseverative errors were counted as regressive errors. Never-reinforced errors were scored when rats made incorrect choice using a rule that has never been rewarded previously. Perseverative errors are considered as a measure of disengaging from an old rule while regressive errors and never-reinforced errors are commonly considered a measure of engaging in and maintaining a new rule.

Counterbalancing of task was conducted to minimize bias in this study: (1) Order of exposure to pairs 1-3 was counterbalanced using a latin square design. For example, pair 1 was used for the SD/CD/CDR in rat 1 while used for the ID/IDR or ED/EDR in rat 2. (2) Half of the rats shifted from odor to medium for the ED (Series 1), and the other half from medium to odor (Series 2). (3) The pairs of exemplars were presented together in a pseudorandom order, with no more than 2 consecutive trials had the same combination of exemplars. For example, if O1 vs M1 and O2 vs M2 in a trial, after no more than two trials, the pattern would be O1 vs M2 and O2 vs M1. (4) The side of baited cup presented in the choice compartments was also varied. Each side was presented for no more than two consecutive trials.

In this study, we took several measures to make the experiment more precise and reliable: (1) We avoided using similar odors, which would presumably increase the difficulty of discrimination and result in increased numbers of trials to criterion. (2) The scented digging medium was renewed every 10 trials in order to avoid scent attenuation. (3) Scent-free baits were chosen to avoid the rat making choice by the scent of the baits instead of the stimuli. (4) The degree of hunger was a potentially confounding factor that may affect the results. If the rat was too hungry, the latency would be shorter and the numbers of errors would be increased compared with those who were relatively full. In this study we housed the rat individually and used the same criteria for diet control. (5) During tests, the rat was drawn back to the waiting compartment repeatedly by the observer and this may cause stress and fear. To enhance adaption, all rats were grasped again and again by their observer for two weeks to reduce stress and fear toward the observer. (6) Rats showed some purposeless behaviors (e.g. rearing, climbing on the cups, sniffing the box walls, etc) after they were put into the test box. The latency was not recorded until animals stopped those behaviors. (7) We defined a digging choice as moving the digging medium with the paws or nose. Contacting or sniffing the cup or medium was not counted. (8) Sessions terminated when the rat refused to dig.

### Statistical analyses

SPSS 15.0 for Windows was used to perform all statistical analyses. Data were analyzed by repeated measures ANOVA with one between-subjects factor (strain/treatment) and one within-subjects factor (stage/error type). Post hoc analysis was conducted for each stage by multivariate ANOVA for all groups followed by LSD test. Violations in sphericity were examined and degrees of freedom were adjusted using the Greenhouse-Geisser and Huynh-Feldt epsilon values for large and small violations respectively. Performance of rats in ID and ED stages, CD and ID stages was compared with paired t-tests. Correlation analyses between the stages were performed. Statistical significance was set at *p *< 0.05. Only trials to criterion were reported in the following analysis [15] as trials and errors to criterion were highly correlated, and analysis of either variable produced the same results.

## Results

**Figure 1:**
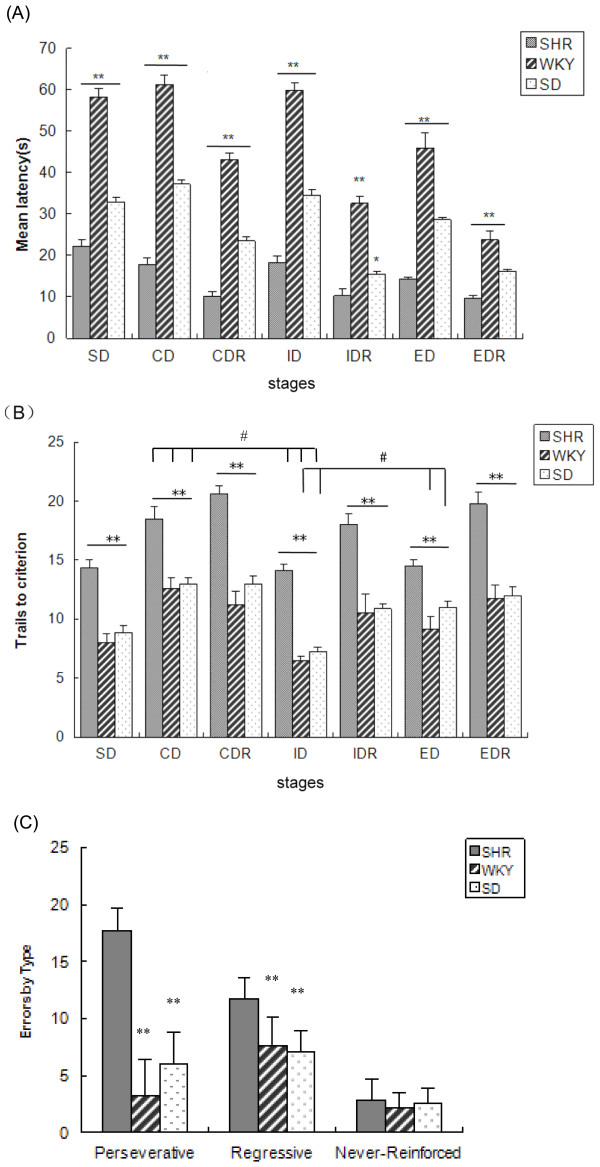
**Strain comparison of the mean latency (A), and the number of trials to criterion (B) for each of the stage during the training. (C) Analysis of the subtypes of errors made by three rats during the whole task**. Values are expressed as mean ± SEM accuracy measured in spontaneous hypertensive rat (SHR), Wistar-Kyoto (WKY) and Sprague-Dawley (SD) strains. *p < 0.05,**p < 0.01 when compared with SHR rats. #*p *< 0.01 compared in CD and ID, ID and ED.

As shown in Figure 1(A), there was a gradual decrease in mean latency from SD to EDR shift for all rats. However, when the rats were faced with new digging medium and new odors in CD, ID and ED shifts, latency was enhanced compared with in the following reversal stages (*p *< 0.001). The SHRs were the quickest responding rats of the 3 strains, with a mean latency of 14.67 s. While the WKY rat, the mean latency of whom was 46.39 s, was the slowest. The mean latency of the SD rat was 26.94 s. The significant main effect of strain [F(2, 21) = 639.636, *p *< 0.001] was observed in the latency. Figure 1 (B) showed the mean trials to criterion of each group at each stage. From the figure we could find significant main effect of strain [F(2, 21) = 114.118, *p *< 0.001] as well as stage [F(6,126) = 21.977, *p *< 0.001], and a non-significant strain by stage interaction in trials [F(12,126) = 1.115, *p *> 0.05]. Post-hoc tests of multiple comparisons revealed that the SHR rats needed significantly more trials to reach criterion compared with control strains (*p *< 0.001) at all stages including simple discrimination, reversal and shifting stages. There was no difference in trials between the WKY and SD strains: no significant main effect of strain in trials [F(1,14) = 2.991, *p *> 0.05], and no significant strains by stages interaction in trials [F(6,84) = 0.298, *p *> 0.05]. There were no significant correlations between SD and ED on trials in SHR (r = -0.245, *p *= 0.558), WKY (r = -0.369, *p *= 0.369) and SD (r = 0.448, *p *= 0.266) rats. The SHR [paired t(7) = 3.379, *p *= 0.012], WKY [paired t(7) = 6.426, *p *= 0.000] and SD rats [paired t(7) = 8.205, *p *= 0.000] had fewer times of trials to criterion at ID compared to CD. Both the WKY [paired t(7) = 2.676, *p *= 0.032] and SD rats [paired t(7) = 4.255, *p *= 0.004] had fewer times of trials to criterion at ID compared to ED. No difference of times of trials between the ED and the ID stages [paired t(7) = 0.456, *p *= 0.662] were found for SHR rats. Figure 1 (C) showed the subtypes of errors made by the 3 rat strains during the whole task. From the figure we could find significant main effect of strain [F(2, 21) = 69.839, *p *< 0.001], error subtype [F(2,42) = 71.194, *p *< 0.001], and strain by error subtype interaction [F(4,42) = 25.121, *p *< 0.01]. Post-hoc tests of multiple comparisons revealed that the SHR rats had significantly more perseverative and regressive errors compared with control strains (*p *< 0.01). This indicates that the SHR has more perseverative responses and is more impaired in maintaining a new rule. No significant difference was found on never-reinforced errors between the SHR and control strains (*p *> 0.05). There was no difference of different types of errors between the WKY and SD strains (*p *> 0.05).

**Figure 2:**
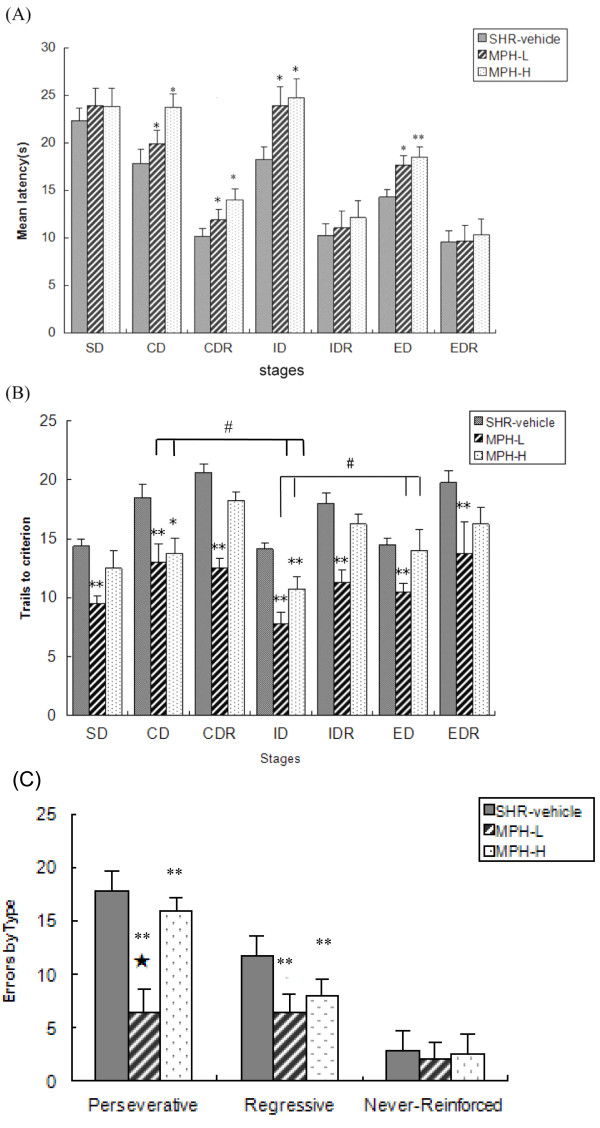
**Effects of treatment with low-dose methylphenidate (2.5 mg/kg) and high-dose methylphenidate (5 mg/kg) on spontaneous hypertensive rat (SHR) strains on the mean latency (A), the number of trials to criterion (B) and the analysis of the subtypes of errors (C) during the attentional set-shifting task**. Values are expressed as mean ± SEM *P < 0.05, **P < 0.01 when compared with vehicle-treated controls. #*p *< 0.01: compared in CD and ID, ID and ED,⋆*p *< 0.01: compared in MPH-H and MPH-L.

Figure 2 (A) showed the mean latency for each of the stages of the MPH and vehicle treated SHRs. Post-hoc test revealed significantly longer latency of the MPH-H group in CD (*p *< 0.05), CDR (*p *< 0.05), ID (*p *< 0.05) and ED (*p *< 0.01) compared with the SHR-vehicle group. Rats in the MPH-L group had significantly longer latency in ID (*p *< 0.05) and ED (*p *< 0.05) than animals in the SHR-vehicle group. In Figure 2 (B), significant main effects of treatment [F(2,21) = 52.174, *p *< 0.001] and stages in trials [F(6,126) = 14.756, *p *< 0.001] and non-significant treatment by stage interaction in trials [F(12,126) = 0.958, *p *= 0.496] were observed. Post-hoc tests revealed significant differences between the SHR-vehicle and MPH-L groups in their abilities to perform overall stages (*p *< 0.01), with the MPH-L group required significantly less trials to reach criterion compared to the SHR-vehicle group. However, only in CD (*p *< 0.05) and ID shift (*p *< 0.01) stages there were significant differences between the SHR-vehicle and the MPH-H groups. Paired-sample T test of the treated SHR showed that more trials were needed in ED than in ID for rats in the MPH-L [t(7) = 3.667, *p *= 0.035] and the MPH-H [t(7) = 3.434, *p *= 0.041] groups. Similarly, more trials were needed in CD than in ID for rats in the MPH-L [t(7) = 4.515, *p *= 0.006] and the MPH-H [t(7) = 5.716, *p *= 0.002] groups. In Figure 2 (C), significant main effects of treatment [F(2,21) = 78.168, *p *< 0.01], error subtype [F(2,42) = 221.635, *p *< 0.01], and treatment by error subtype interaction [F(4,42) = 23.44, *p *< 0.01] were observed. Post-hoc tests revealed significant difference in perseverative and regressive errors between the MPH-L and SHR-vehicle groups (*p *< 0.01). Significant difference in perseverative errors was found between MPH-L and MPH-H groups (*p *< 0.01).

## Discussion

In Experiment 1, we observed poorer performance of the SHR overall on attentional set-shifting task than the WKY and SD rats, which suggested that the SHR was impaired in discrimination learning, reversal learning and attentional set-shifting. Based on these results, we believe that impulsivity, perseverative responding and impairment in maintaining attention could all contribute to the overall impairment on attentional set-shifting tasks of the SHRs.

In the ASST, the latency changes can be understood as either different in speed, accuracy trade off or simply alterations in locomotor activity [35]. If a rat with long latencies shows few trials to criterion, it might indicate a good accuracy trade off strategy. If a rat with short latencies shows more trials to criterion, then it might indicate impulsiveness of the rat. In this test, the SHRs generally had more trials than the WKY and SD rats in a series of successive discriminations, while the latencies of the SHR were significantly shorter than the other two strains in the whole test. This implied that the SHR was the most impulsive rat of the three strains. The result was just consistent with what we observed during the test. When the sliding door was removed, the SHR dug the cups immediately without any stagger, seldom trading off between the two cups before digging. With impulsiveness, the SHR was difficult to have self-control and make a right decision, thus they had much more errors and trials than the other two strains. Impulsive behavior occurred at all stages, so the SHR had impaired performance at all stages.

Our finding appears consistent with Kantak [6] et al.'s study which found that the SHRs performed more poorly than the WKY rats, but contradictory to the other published study to examine set-shifting in SHRs by Chess AC et al. [14] which reported that SHRs were faster than Wistar rats in shifting to the opposite discrimination when there was 1 or 2 days between the initial discrimination and the shift. We used the WKY and SD rats and Kantak [6] et al. used WKY rats as control stains, while Chess AC et al. used the Wistar. The difference of strains of the control groups may underlie some of the discrepancies in results and interpretations in studies [36]. Given the importance of set-shifting for understanding ADHD, further examination of this issue may be needed.

Another reason is that the SHR has more perseverative response in shifting. This study found that the SHRs made significantly more perseverative errors than the other 2 control strains, which indicates that it is difficult for the SHRs to disengage from previously relevant but currently irrelevant strategy during attentional set-shifting task. It provided an index of poor behavioural and cognitive flexibility in SHR. Increased perseverative responses resulted in more series of response at all stages. What's more, in this study, the SHRs made significantly more regressive errors than the other 2 control strains, which indicated that they might be impaired in maintaining attention. This may be the reason of poorer performance of the SHR overall on attentional set-shifting task than the WKY and SD rats.

In ASST, It is important to form attentional set firstly before shifting attentional set. A rat that forms an attentional set should master the ID stage more rapidly (fewer trials to criterion) than the original discrimination (CD stage) and poorer performance on ED compared with ID (fewer trials to criterion in ID stage than in ED stage). In our test, we observed significantly fewer trials to criterion in ID stage than in CD stage and ED stage in WKY and SD rats, which suggested that they could form attentional-set successfully. Meanwhile, it indicated that the paradigm we used here could serve as a valuable test paradigm for periadolescent rats. However, the task performance in ID stage was not improved in SHRs compared with in ED stage, which suggest that it was difficult for the SHR to acquire an attentional set by a series of successive trials. The failure of forming attentional set of the SHR may be due to their defect of maintaining attention [37] or dramatic increase in perseverative responding at the shifting [38]. Garner [39] presumed that ED was dependent on the repeated presentations of the relevant dimensions. The SHR may need more repeated trials to set up an attentional set. Without the ability to form attentional set, the ED stage may not be a suitable test to evaluate the ability of set-shifting. Thus, it was not possible to conclude with certainty that attentional set-shifting was impaired in the SHRs compared with the WKY and SD rats. However, Garner [39] considered the perseverative responding as one of the reasons relevant to impaired set-shifting. In this test, the SHR strain was more inclined toward perseverative responding than other strains. So, the SHR rats may have a poorer ability of set-shifting compared to other strains. Previous studies indicate that the increase in perseverative errors during set-shifting is related to the dysfunction of medial PFC. Abnormality in the dorsomedial striatum may underlie the increase of regressive errors [31,32]. Thus the SHRs may have impairment of the medial PFC and the dorsomedial striatum.

In our tests, there was no significant correlation in performance between SD and ED among all the rats. The performance in SD could not be the predicted performance in the final ED shift in this test. Our result was contradictive from the study of Colacicco G [15] et al., which showed that performances in these two tasks were significantly correlated in mice. The explanation might be that our rats were given formal training on discrimination learning prior to the onset of testing.

In the present study, we found that the performance of the WKY strain in the ASST was similar to that of the SD strain at all stages. This finding supported the continued use of WKY as a control for SHR to investigate the ability of behavioural and cognitive flexibility in ADHD. Moreover, it suggested that SD rats may be another useful additional reference strain for SHR.

From the results of Experiment 1, we observed that like individuals with ADHD, the SHRs may also have poor performance at attentional set-shifting task compared with the WKY and SD rats. Clear delineation of the effects of MPH upon attentional set-shifting deficits in ADHD would enhance understanding of the effects of drug treatment. In Experiment 2, lower dose (2.5 mg/kg) and higher dose (5.0 mg/kg) of MPH were administered to rats to investigate the dose effect of MPH on acquiring, maintaining and shifting attentional set of the SHRs. The results revealed effects of MPH of different doses on attentional set-shifting. The SHRs who received lower-dose MPH showed improved performance at all stages than the SHR-vehicle group especially at ED stage. This indicated that lower-dose MPH could improve PFC-dependent cognitive ability to shift attentional focus of the SHR. Furthermore, after treatment of lower dose MPH, the SHR could set up attentional set before set shifting (fewer trials to criterion at ID stage than at CD and ED stages). The reason maybe that lower-dose MPH could ameliorate impulse, reduce perseverative response and improve ability of maintaining attention as rats in the MPH-L group had increased latency, reduced perseverative and regressive errors than the SHR- vehicle group. These results were in consistent with previous clinical reports [40] which showed that lower dose treatment of MPH on ADHD could reduce movement and impulsivity and enhance cognitive function, including sustained attention and working memory. The performance of higher-dose MPH treated SHR improved significantly only in CD stage, ID stage and regressive errors, also formed the attentional set (fewer trials to criterion in ID stage than in CD stage and ED stage), but it was noteworthy that the perseverative errors and other performance of reversal (CDR, IDR, EDR) stages and set-shifting (ED) stage were not changed in this group compared to the SHR-vehicle group. A possible explanation may be that the higher dose of MPH did not have a significant effect on perseverative responding and may have even facilitated perseverative responding to some degree. Arnsten [20] found that higher doses of MPH induced a perseverative profile of errors in that the rats continued choosing the same incorrect arm of the maze in delayed alternation performance. Similar to rodents, clinicians also considered that higher doses of MPH could induce perseverative thinking in patients [41]. Thus, the lower dose of MPH may be more effective than the higher dose in improving the ability of attentional set-shifting of the SHR.

Catecholamines in the PFC play a pivotal role in the cognition-enhancing and therapeutic actions of MPH. At higher doses, MPH may produce huge and widespread increases in extracellular levels of noradrenaline (NE) and dopamine (DA) and induce excessive catecholamine receptor stimulation throughout the brain, which can impair PFC cognitive function. In contrast, lower doses of MPH, which have behavioral-calming and cognition-enhancing functions, can exert regionally restricted actions, minimal elevated extracellular catecholamine levels and enhanced catecholamine receptor actions [20,40] preferentially within the PFC [19]. Studies showed that lesions of dorsolateral PFC in primates [42] and of the corresponding medial PFC in rats and mice [8] induced impairment on the attention set-shifting, and lesions to the orbitofrontal cortex (OFC) in primates and rodents impaired reversal learning [43,44]. By single photon emission computerized tomography (SPECT), Lee JS [45] found that MPH could improve ADHD symptoms by normalizing the OFC activity. Imaging studies have shown that more efficient dorsolateral PFC activity can be found following MPH treatment which is considered to improve PFC cognitive function [17]. Thereby, we assume that lower dose MPH may preferentially increase catecholamine neurotransmission within the PFC and enhance the ability of reversal learning and attentional set-shifting. In contrast, higher dose MPH may induce excessive catecholamine receptor stimulation throughout the brain and fail to ameliorate the performance on reversal and shifting set.

## Conclusions

In conclusion, the present study revealed deficits in acquiring, sustaining and shifting attentional set and reversal learning in the SHRs, a genetic animal model of ADHD. MPH treatment of ADHD in the SHRs may ameliorate deficits in attentional set-shifting differently, and lower doses were more effective than higher doses. We provided the first evidence of the dose-dependent effect of MPH on attentional set-shifting performance in SHRs.

## Abbreviations

ADHD: Attention deficit/hyperactivity disorder; SHR: Spontaneously hypertensive rat; WKY: Wistar-Kyoto; SD: Sprague-Dawley; ASST: Attentional set-shifting task; MPH: Methylphenidate; CANTAB: Cambridge Neuropsychological Test Automated Battery; TMT: Trail Making Test; WCST: Wisconsin Card Sorting Task; SD: Simple discrimination; CD: Compound discrimination; CDR: Reversal of compound discrimination; ID: Intradimensional shift; ED: Extradimensional shift; PFC: Prefrontal Cortex; SPECT: Single photon emission computerized tomography; NE: Noradrenaline; DA: Dopamine.

## Competing interests

The authors declare that they have no competing interests. The authors alone are responsible for the content and writing of the paper.

## Authors' contributions

Ai-hua Cao carried out the behavior study and performed the statistical analysis. Lin Yu, Yu-wei Wang, Jun-mei Wang, and Le-jin Yang participated in the behavior study and helped to draft the manuscript. Ge-fei Lei conceived of the study and drafted the manuscript. All authors read and approved the final manuscript.
